# Cardiac-specific overexpression of Ndufs1 ameliorates cardiac dysfunction after myocardial infarction by alleviating mitochondrial dysfunction and apoptosis

**DOI:** 10.1038/s12276-022-00800-5

**Published:** 2022-07-11

**Authors:** Bingchao Qi, Liqiang Song, Lang Hu, Dong Guo, Gaotong Ren, Tingwei Peng, Mingchuan Liu, Yexian Fang, Chunyu Li, Mingming Zhang, Yan Li

**Affiliations:** 1 Department of Cardiology, Tangdu Hospital, Fourth Military Medical University, Xi’an, 710038 People’s Republic of China; 2 Department of Pulmonary and Critical Care Medicine, Xijing Hospital, Fourth Military Medical University, Xi’an, 710032 People’s Republic of China

**Keywords:** Heart failure, Myocardial infarction, Myocardial infarction

## Abstract

Myocardial infarction (MI) is the leading cause of premature death among adults. Cardiomyocyte death and dysfunction of the remaining viable cardiomyocytes are the main pathological factors of heart failure after MI. Mitochondrial complexes are emerging as critical mediators for the regulation of cardiomyocyte function. However, the precise roles of mitochondrial complex subunits in heart failure after MI remain unclear. Here, we show that NADH:ubiquinone oxidoreductase core subunit S1 (Ndufs1) expression is decreased in the hearts of heart failure patients and mice with myocardial infarction. Furthermore, we found that cardiac-specific Ndufs1 overexpression alleviates cardiac dysfunction and myocardial fibrosis in the healing phase of MI. Our results demonstrated that Ndufs1 overexpression alleviates MI/hypoxia-induced ROS production and ROS-related apoptosis. Moreover, upregulation of Ndufs1 expression improved the reduced activity of complex I and impaired mitochondrial respiratory function caused by MI/hypoxia. Given that mitochondrial function and cardiomyocyte apoptosis are closely related to heart failure after MI, the results of this study suggest that targeting Ndufs1 may be a potential therapeutic strategy to improve cardiac function in patients with heart failure.

## Heart attack: preventing subsequent heart failure

Boosting a key energy-production enzyme can help cardiomyocytes, the cells that make the heart contract, recover after a heart attack. A heart attack interrupts blood flow, damaging cardiomyocytes; these damaged cardiomyocytes can later lead to heart failure. Components of mitochondria, the cellular powerhouses, are known to be critical to cardiomyocyte function, but their role in heart failure remained unclear. Yan Li and Mingming Zhang at the Fourth Military Medical University, Xi’an, China, and coworkers examined publicly available data showing which mitochondrial components are altered after a heart attack. Levels of enzyme Ndufs1, one of the largest components, were significantly decreased. Boosting Ndufs1 in hearts during the healing phase improved heart function in mice. Boosting Ndufs1 levels repaired damaged mitochondria, which in turn improved cardiomyocyte function. These results may help to find new treatments following a heart attack.

## Introduction

Globally, cardiovascular diseases are the most common cause of death^1^. Heart failure, the end-stage phenotype in most cardiovascular diseases, has a poor long-term prognosis, with a 5-year mortality rate exceeding that of some common cancers, such as prostate cancer in men and breast cancer in women^2,3^. In the last two decades, myocardial infarction has been the most common cause of heart failure^4^. During MI, the sudden interruption or reduction of the blood supply predisposes the remaining surviving cardiomyocytes to death or dysfunction^5^. Post-MI apoptosis and myocardial fibrosis are critical pathological features of adverse myocardial remodeling and predominant causes of heart failure^6–8^. Although current advances in interventional and pharmacological strategies have yielded better clinical outcomes for patients with MI, as indicated by the decline in the in-hospital and 1-year mortalities, the incidence and mortality of post-MI heart failure remain high^9–11^. Therefore, it is essential to elucidate the mechanisms of post-MI heart failure and identify new therapeutic targets.

Cardiac contraction and diastole require a constant supply of ATP, which is derived from oxidative phosphorylation in the mitochondria. Mitochondrial complex I, the initiating and largest enzyme of the mitochondrial respiratory chain, is responsible for electron transfer from NADH to coenzyme Q10 and is coupled to proton translocation from the matrix to the intermembrane space^12^. Mammalian complex I comprise 45 subunits, and 7 of the 14 core subunits are encoded by nuclear genes^13,14^. Ndufs1, the largest core subunit encoded by nuclear genes, is a part of the eight iron-sulfur chains and is responsible for the oxidation of NADH^15,16^. Proton translocation across the inner mitochondrial membrane generates an electrochemical gradient that drives ATP production in complex V. As a byproduct of simultaneous ATP production, ROS are also generated in several mitochondrial complexes, mainly complex I^17,18^. A previous study demonstrated that Ndufs1 knockdown in neurons resulted in impaired oxygen consumption and increased mitochondrial ROS, and upregulated Ndufs1 expression in astrocytes also decreased ROS generation^19^. In skin fibroblasts obtained from patients with the Ndufs1 mutation, a high level of oxidative stress was found, accompanied by a decrease in complex I activity, impaired oxygen consumption, and increased glycolysis^20^. As the starting point of the mitochondrial electron transport chain (ETC), Ndufs1 is a dual “engine” for ATP and ROS production and plays a vital role in metabolic reprogramming, oxidative stress, and cell apoptosis in several diseases^20–22^. However, whether Ndufs1 plays a role in the pathological processes of heart failure after MI is unknown.

The present study was designed to determine whether and how Ndufs1 influences myocardial injury. We first upregulated Ndufs1 expression in mouse hearts by the adeno-associated virus serotype 9 (AAV9) method to clarify the effect of Ndufs1 on cardiac function in mice post-MI. Furthermore, the protective mechanism of Ndufs1 was explored through gene regulation and functional experiments on cardiomyocytes.

## Materials and methods

### Animals

All experimental mice had a C57BL/6 J background and were obtained from the animal center at Fourth Military Medical University. All animal experimental designs and protocols followed the Guide for the Care and Use of Laboratory Animals and were approved by the Fourth Military Medical University Committee on Animal Care. Mice were maintained in a specific pathogen-free environment with a standard temperature (20–23 °C) and a 12 h light-dark cycle. Experimental mice were randomly grouped, and blinding was performed for all in vivo experiments and subsequent assessments.

### Mouse MI model

Male mice (8–9 weeks) were anesthetized with isoflurane (1–2%), and myocardial infarction was induced as described previously^23^. In brief, the mouse hearts were quickly squeezed out of the thoracic cavity via the left thoracic incision. To induce myocardial infarction, we used a silk suture (6-0) to ligate the left anterior descending (LAD) coronary artery. The whitening of the ischemic area and changes in the electrocardiogram were important indicators of the success of the procedure. Sham-operated control mice were subjected to identical surgical procedures without coronary artery ligation.

### Human heart samples

Non-failing (EF > 60%) heart samples and failing (EF < 40%) heart samples were obtained from patients who received coronary bypass surgery. This study was approved by the Ethics Committee of Tangdu Hospital, Fourth Military Medical University (No. 202103-12). All patients were fully aware of the nature of the study and expressed their willingness to participate through an informed consent form.

### Isolation of neonatal rat cardiomyocytes (NRCMs) and HL-1 cell culture

NRCMs were isolated from 1–2-day-old Sprague-Dawley rats. In brief, the cardiac tissue was washed twice with PBS to remove blood. Next, it was cut into small pieces and digested with type I collagenase solution (1 mg/ml, Thermo Fisher Scientific, Waltham, MA, USA) five to six times. Finally, the digestion process was terminated with the addition of a complete medium. Since fibroblasts and cardiomyocytes have different attachment times, the differential attachment method was used to remove fibroblasts as much as possible. The primary cardiomyocytes were cultured in complete medium for the initial 48 h. The HL-1 cell line, a mouse cardiac muscle cell line, is derived from the AT1 mouse atrial cardiomyocyte tumor lineage^24^. HL-1 cells were used for ChIP assays and cellular functional assays. Before induction of hypoxia in cells, the medium was replaced with a sugar-free and serum-free medium to simulate nutrient deprivation. Moreover, the cells were placed in a hypoxic chamber (5% CO_2_ and 95% N_2_) to continue culturing. Hypoxia pre-experiments were performed to explore the tolerance of different cells to hypoxia. The hypoxia time was 9 h for NRCMs and 3 h for HL-1 cells.

### Downloading and analysis of public datasets

RNA-seq data for human heart tissues were downloaded from the GEO database (www.ncbi.nlm.nih.gov/geo). The GSE161472 dataset is a high-throughput sequencing dataset for studying heart failure with reduced ejection fraction (HFrEF). RNA samples were isolated from the four chambers of non-failing (NF) hearts and hearts with HFrEF. The genetic composition data of the mitochondrial complex were obtained from the Mitopathways annotated in the M3.0 database (http://www.broadinstitute.org/mitocarta)^25^.

### Quantitative real-time PCR (qRT-PCR)

Total RNA was isolated from heart tissues and cells using RNAiso Plus (TaKaRa, Otsu, Japan) following the manufacturer’s protocols. Reverse transcription was performed using the PrimeScript RT reagent kit (TaKaRa). qRT-PCR was performed using TB Green Premix Ex Taq II (TaKaRa) on a CFX96 real-time PCR detection system (Bio-Rad, Hercules, CA, USA). The cycling parameters were as follows: first step at 95 °C for 30 s, a second step at 95 °C for 5 s, and 60 °C for 30 s; the second step was repeated for 40 cycles. The primer sequences are shown in Supplementary Table 1. β-actin or GAPDH was used as the internal control.

### Western blotting

Cardiac tissues below the ligature and cultured cardiomyocytes were homogenized and lysed with RIPA buffer (Beyotime, China). Proteins (25 μg) were separated on SDS-PAGE gels, transferred to polyvinylidene fluoride (PVDF) membranes, blocked with 5% milk, and incubated overnight at 4 °C with primary antibodies. Details of all antibodies are shown in Supplementary Table 2. After the membranes were incubated with HRP-conjugated secondary antibody for 1 h at room temperature, the bands were detected on a chemiluminescence system (Bio-Rad). The immunoblot band intensity was analyzed and quantified using LabImage software (Bio-Rad). β-actin or GAPDH was used as the internal control.

### Histological staining

Mouse heart samples were fixed overnight with 4% paraformaldehyde, embedded in paraffin, and cut into 5-μm-thick sections. Paraffin sections were stained with hematoxylin and eosin (H&E), and the morphology of cardiac tissue was observed. Masson’s trichrome staining was performed to assess the cardiac collagen content and determine the infarct size. Infarct size was assessed as the total infarct circumference divided by the total left ventricle circumference. For wheat germ agglutinin (WGA) staining, freshly dissected heart samples were embedded in an OCT embedding matrix and were cut into 10-μm-thick sections. Sections were incubated with WGA solution (10 µg/ml) for 1 h in the dark. Images were obtained with a confocal laser scanning microscope (Nikon A1R MP + Confocal Microscope; Nikon, Tokyo, Japan) and analyzed with ImageJ software (Rawak Software, Stuttgart, Germany). For triphenyl tetrazolium chloride (TTC) staining, mouse hearts were obtained 24 h post-MI, and four 1.0 mm thick sections were continuously cut from the ligature to the apex. The sections were then incubated with 1% TTC solution (Sigma-Aldrich, St. Louis, MO, USA) and photographed. Images were captured and analyzed using ImageJ software.

### Echocardiography

Transthoracic echocardiography was performed in the M-mode using the Vevo 2100 ultrasound system (VisualSonics, Inc., Toronto, ON, Canada). Mouse chest hairs were removed with depilatory cream before the experiment. Then, the mice were anesthetized with 2% isoflurane and placed on a warm heating pad. Cardiac function was measured in M-mode, and each mouse heart was imaged in both the long-axis and short-axis views. The left ventricular systolic internal dimension (LVIDs) and left ventricular diastolic internal dimension (LVIDd) were obtained by indirect measurements using Vevo 2100 software (VisualSonics, Inc.). The ejection fraction (EF) was obtained by further analysis. All measurements were performed by a researcher who was blinded to the experimental treatments.

### AAV9 vector construction and administration

Adeno-associated virus serotype 9 (AAV9), under the control of a cardiac troponin T promoter (cTnT), was used to overexpress the target gene in the animal model. AAV9-cTnT-Ndufs1 and negative control AAV9-cTnT-Ctrl were constructed by GeneChem Technology (Shanghai, China). For upregulated Ndufs1 expression in mice, after the mice (4 weeks old) were anesthetized with 2% isoflurane, 10^11^ viral particles were administered by intrathoracic injection. AAV9-Ctrl was injected using the same method as in the control. Four weeks after the injection, heart samples were collected to detect protein expression, and an MI operation was performed.

### Upregulation and downregulation of target gene expression in cardiomyocytes

Adenoviral vectors carrying Ndufs1 (Ad-Ndufs1) and adenoviral empty vector (Ad-EV) constructs were generated by GenePharma Company (Shanghai, China). Adenoviral vectors carrying Sp1 (Ad-sp1) and Ad-EV were purchased from Hanbio Technology (Shanghai, China). After infection with adenovirus for 48 h, protein expression was detected. Ndufs1-specific small interfering RNA (siNdu), Sp1-specific small interfering RNA (siSp1), and control small interfering RNA (siCtrl) were designed and synthesized by GenePharma Company. siRNA was transfected into cardiomyocytes using Lipofectamine RNAiMAX (Invitrogen, Waltham, MA, USA). The sequences of the siRNAs are shown in Supplementary Table 3.

### Transmission electron microscopy (TEM)

Myocardial mitochondrial cristae morphology was observed by TEM, and the protocol to prepare heart samples was as described previously^26^. Mouse left ventricles were cut into longitudinal strips along fiber orientation with a width of 1–2 mm. Samples were prefixed with 4% glutaraldehyde for 24 h at 4 °C and then postfixed with 1% osmium tetroxide for 2 h at 4 °C. After dehydration and embedding, the samples were cut into ultrathin sections and stained with uranium acetate and lead citrate. All images were obtained with a transmission electron microscope (JEM-1230, JEOL, Ltd., Tokyo, Japan).

### Measurement of mitochondrial respiratory function

Cardiomyocytes from one neonatal rat, on average, were laid on a 24-well seahorse assay plate (Agilent Seahorse Bioscience, Santa Clara, CA, USA). After 48 h of culture, siRNA or adenovirus treatment was transfected for 48 h, followed by a mitochondrial oxygen consumption rate (OCR) assay using an XF24 Extracellular Flux Analyzer (Agilent Seahorse Bioscience), as described previously^27^. The working concentrations of the four reagents were as follows: 1 µM oligomycin, 1 µM FCCP, 1 µM rotenone, and 0.5 µM antimycin A. OCR data were obtained and analyzed using the XF Cell Mito Stress Test Generator software (Agilent Seahorse Bioscience).

### Mitochondrial complex I activity assay

Mitochondrial complex I activity was measured using a Complex I Enzyme Activity Assay Kit (Abcam) following the manufacturer’s instructions.

### DHE staining and MitoSOX staining

Total myocardial ROS levels were measured by incubating the paraffin sections in dihydroethidium (DHE) solution at 37 °C for 30 min. Mitochondrial ROS levels were detected using the fluorescent probe MitoSOX (Invitrogen) following the manufacturer’s protocols. All images were obtained with a confocal laser scanning microscope (Nikon A1R MP + Confocal Microscope) and analyzed with ImageJ software.

### TUNEL staining

Heart tissue apoptosis was measured using a TUNEL assay kit (Sigma-Aldrich). The proportion of apoptotic nuclei was calculated as the total number of TUNEL-positive nuclei divided by the total number of DAPI-positive nuclei. On average, six random fields were analyzed per paraffin section.

### Chromatin immunoprecipitation (ChIP)

ChIP assays were performed using the SimpleChip Plus Enzymatic ChIP kit (Cell Signaling Technology, Danvers, MA, US) following the manufacturer’s instructions. In brief, protein and DNA in HL-1 cells were crosslinked with 1% formaldehyde. Chromatin was digested into DNA fragments of appropriate length (150-900 bp) using micrococcal nuclease and ultrasonic treatment. Enough chromatin was used for immunoprecipitation and incubated with a Sp1 antibody. Normal IgG was used as the negative control.

### Reduced glutathione (GSH) and glutathione peroxidase (GPx) assay

Cardiomyocyte GSH levels were measured using the Micro Reduced Glutathione Assay Kit (Solarbio, China) following the manufacturer’s instructions. Cardiomyocyte GPx activity was measured using the Total Glutathione Peroxidase Assay Kit with NADPH (Beyotime, China) following the manufacturer’s instructions.

### Statistics

All data were analyzed using GraphPad Prism 8 (GraphPad Software, La Jolla, CA, USA) and are presented as mean ± SEM. Unpaired Student’s *t*-test was used to analyze the differences between the two groups. One-way analysis of variance (ANOVA) was performed with a Bonferroni post hoc test for multiple group comparisons. *p* values <0.05 were considered statistically significant.

## Results

### Ndufs1 expression is decreased in the myocardium of heart failure patients and post-MI mice

**Fig. 1:**
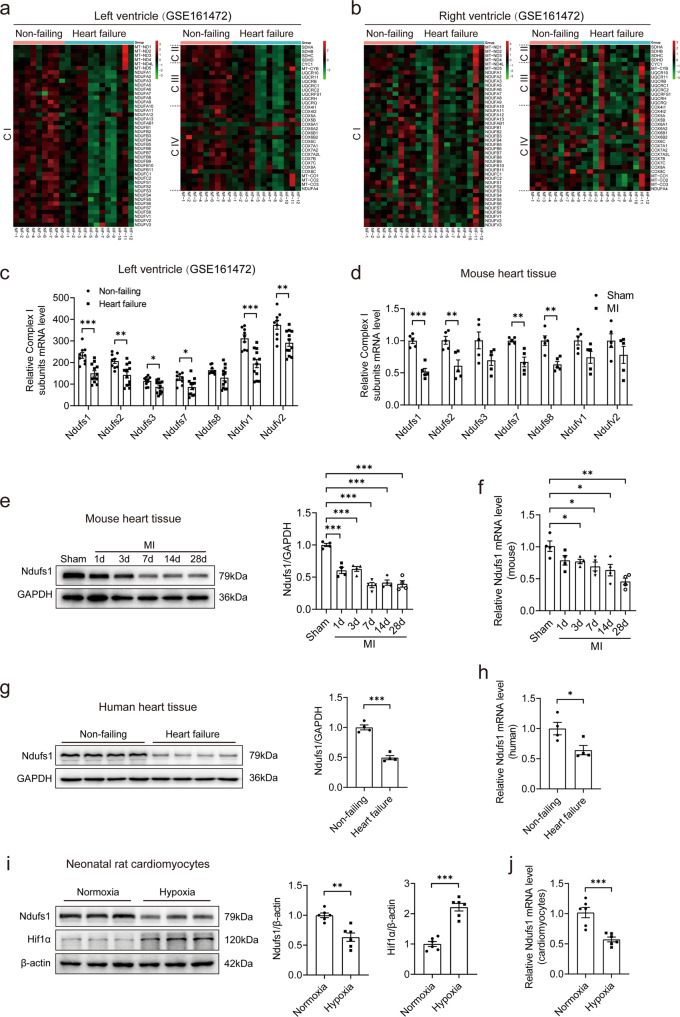
Ndufs1 expression is decreased in the myocardium of heart failure patients and post-MI mice. **a**, **b** Heatmap of RNA-seq analysis for mitochondrial genes belonging to complex I (C I), complex II (C II), complex III (C III), and complex IV (C IV) in the left ventricle samples and right ventricle samples of non-failing subjects (*n* = 9) and heart failure patients (*n* = 12). Red indicates high expression; green indicates low expression. The larger the value is, the more obvious the increase/decrease is. **c** Mitochondrial complex I subunit mRNA levels in the left ventricle of non-failing subjects (*n* = 9) and heart failure patients (*n* = 12) based on RNA-seq data. **d** qRT-PCR analysis of mitochondrial complex I subunit mRNA levels in the hearts of the sham mice and mice on the 28th-day post-MI (*n* = 5 per group). **e**, **f** Western blotting and qRT-PCR analyses of Ndufs1 expression in mouse heart tissues at the indicated time points post-MI (*n* = 4 per group). **g**, **h** Western blotting and qRT-PCR analyses of Ndufs1 expression in human heart tissues (*n* = 4 per group). **i**, **j** Western blotting and qRT-PCR analyses of Ndufs1 expression in NRCMs with or without hypoxia (*n* = 6 per group); Hif1a was used as a marker of hypoxia. Data were presented as mean ± SEM. Statistical significance was assessed by one-way ANOVA. *p < 0.05, **p < 0.01, ***p < 0.001.

To investigate the changes in subunits of mitochondrial complexes in the myocardium of heart failure patients, we examined the publicly available RNA sequencing data obtained from four chambers of non-failing (NF) hearts and hearts with heart failure (HF). Compared with NF human hearts, multiple mitochondrial complex subunits had lower levels in the hearts with HF (Fig. 1a, b and Supplementary Fig. 1a, b). Interestingly, there were also significant differences in the four heart chambers of the HF patients (Fig. 1a, b and Supplementary Fig. 1a, b). Given that mitochondrial complex I is the initiator of the ETC (NADH pathway) and has an intricate structure, we further analyzed the seven core subunits of complex I encoded by the nucleus from both human and mouse samples. The mRNA levels of Ndufs1, Ndufs2, and Ndufv2 were significantly decreased in the left ventricle (LV) and left atrium (LA) of the HF patients compared with those of the NF samples (Fig. 1c and Supplementary Fig. 1c). These changes were not completely consistent in the right heart of the HF patients (Supplementary Fig. 1d, e). Furthermore, we found that the mRNA levels of Ndufs1, Ndufs2, Ndufs7, and Ndufs8 were significantly decreased in the mice with MI compared with the sham mice (Fig. 1d). Given that Ndufs1 is the largest subunit of complex I, it piqued our interest. Western blotting showed that Ndufs1 protein expression was reduced on the 1st-day post-MI and dropped to the lowest point (0.4-fold) on the 7th day; thereafter, the expression remained low until 28 days (Fig. 1e). qRT-PCR data at different time points post-MI showed similar results, but the lowest level (0.5-fold) of Ndufs1 was found on the 28th-day (Fig. 1f). Immunohistochemistry results also confirmed a significant decrease in the Ndufs1 protein level of myocardial tissue on the 28th-day post-MI compared with that of the sham-treated mice (Supplementary Fig. 1f). In heart tissues obtained from human HF patients, both the protein and mRNA levels of Ndufs1 were significantly lower than those in the non-failing group (Fig. 1g, h). In addition, consistent with the findings in vivo, hypoxia significantly decreased the protein and mRNA levels of Ndufs1 in isolated NRCMs (Fig. 1i, j). Given the reduced expression of Ndufs1 in both heart tissue samples and NRCMs, we hypothesized that Ndufs1 is strongly involved in the cardiac pathology of myocardial infarction and heart failure.

### Cardiac-specific overexpression of Ndufs1 fails to alleviate cardiac dysfunction in the acute phase of MI

**Fig. 2:**
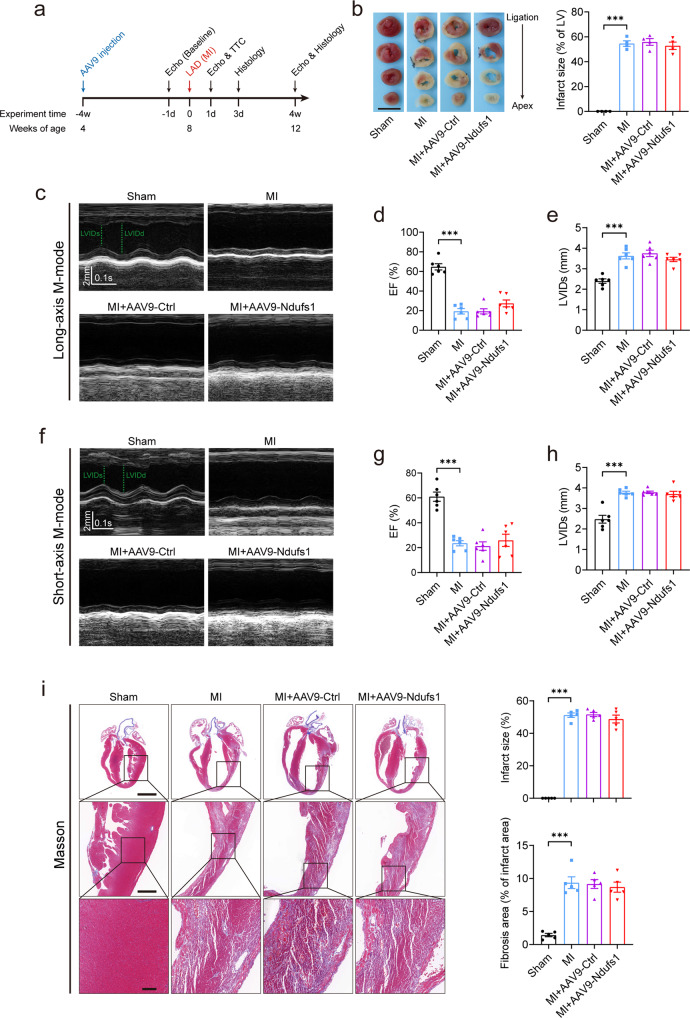
Cardiac-specific overexpression of Ndufs1 fails to alleviate cardiac dysfunction in the acute phase of MI. **a** Timeline of the in vivo experimental design for Ndufs1 gene therapy. **b** Infarct size measured by TTC staining at 24 h post-MI (*n* = 4 per group); scale bar = 5 mm. **c** Representative long-axis M-mode echocardiograph images on the 1st-day post-MI. LVIDs left ventricular systolic internal dimension, LVIDd left ventricular diastolic internal dimension. **d**, **e** Echocardiograph analysis in long-axis M-mode (*n* = 6 per group). EF ejection fraction. **f** Representative short-axis M-mode echocardiographic images on the 1st-day post-MI. **g**, **h** Echocardiograph analysis in the short-axis M-mode (*n* = 6 per group). **i** Representative Masson trichrome staining images and quantification of infarct size/fibrotic area on the 3rd-day post-MI (*n* = 5 per group); upper scale bar = 2 mm, middle scale bar = 500 µm, lower scale bar = 100 µm. Data were presented as mean ± SEM. Statistical significance was assessed by one-way ANOVA. *p < 0.05, ***p < 0.001.

To verify the functional role of Ndufs1 in gene therapy, we injected AAV9-cTnT-Ndufs1 or AAV9-cTnT-Ctrl constructs into the thoracic cavity of C57BL/6 J mice at four weeks of age (Fig. 2a and Supplementary Fig. 2a). Significant overexpression of Ndufs1 was observed in the hearts of the mice injected with AAV9-Ndufs1 (Supplementary Fig. 2b). Four weeks after AAV injection, cardiac-specific overexpression of Ndufs1 showed no significant effect on the body weights of the mice (Supplementary Fig. 2c). Cardiac function and left ventricular structure were similar in all groups at 4 weeks after AAV injection, as evidenced by the EF and LVIDs (Supplementary Fig. 2d–i). The timeline of the experimental design is shown in Fig. 2a. MI surgery was performed four weeks after the AAV injection. In the acute phase of MI (24 h post-MI), the MI group showed a significantly enhanced infarct size compared with the sham group through TTC staining analysis (Fig. 2b). However, myocardial injury to a similar extent was observed by a comparable infarct size in the AAV injection groups (Fig. 2b). Echocardiography in the acute phase of MI was performed on the first-day post-MI, and both long-axis M-mode and short-axis M-mode were executed. Cardiac dysfunction and left ventricular dilatation in the AAV9-Ndufs1 group were not alleviated, as indicated by the similar EF and LVIDs values in the AAV9-Ctrl group (Fig. 2c–h). On the third day post-MI, the cardiac Ndufs1-overexpressing mice displayed a comparable infarct size and myocardial fibrotic level compared with the AAV9-Ctrl mice (Fig. 2i). Similarly, the cardiac and pulmonary hypertrophic adaptations in response to hypoxia were significant in the mice with MI compared with the sham-treated mice; however, no significant differences were observed in the AAV-injected mouse groups (Supplementary Fig. 3a–e). These data suggested that cardiac-specific overexpression of Ndufs1 exerted no significant protective effect on cardiac function and myocardial fibrosis in the acute phase of MI.

### Cardiac-specific overexpression of Ndufs1 effectively alleviates cardiac dysfunction and myocardial fibrosis in the healing phase of MI

**Fig. 3:**
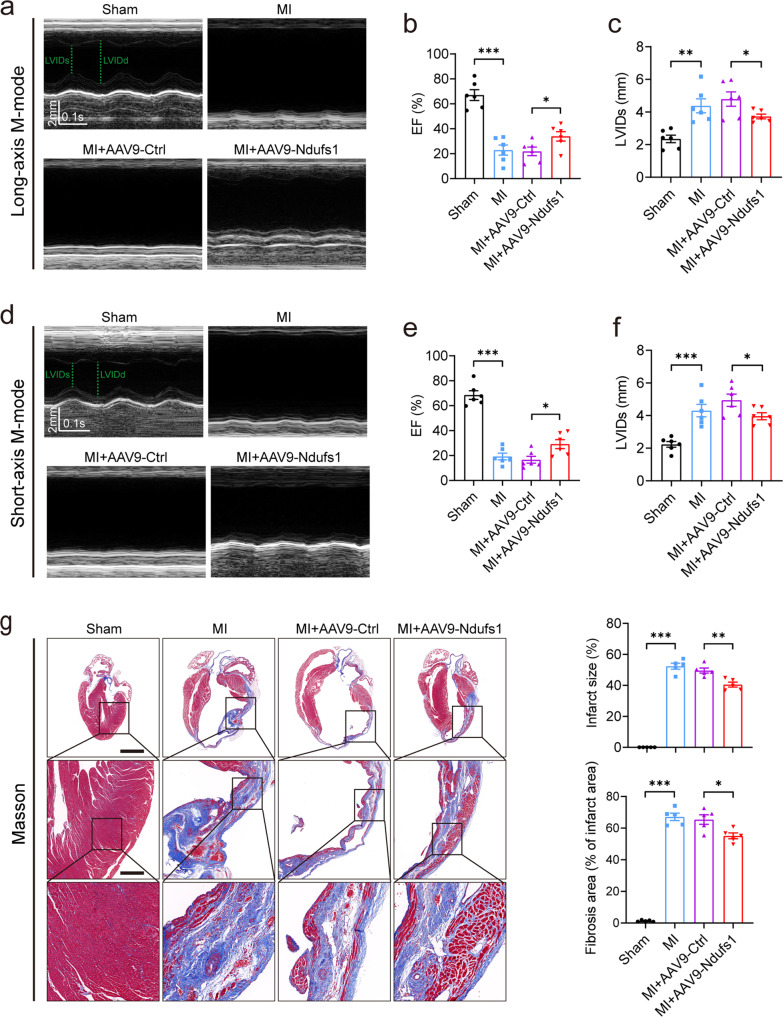
Cardiac-specific overexpression of Ndufs1 effectively alleviates cardiac dysfunction and myocardial fibrosis in the healing phase of MI. **a** Representative long-axis M-mode echocardiograph images on the 28th-day post-MI. **b**, **c** Echocardiograph analysis in the long-axis M-mode (*n* = 6 per group). **d** Representative short-axis M-mode echocardiograph images on the 28th-day post-MI. **e**, **f** Echocardiograph analysis in the short-axis M-mode (*n* = 6 per group). **g** Representative Masson trichrome staining images and quantification of infarct size/fibrotic area on the 28th-day post-MI (*n* = 5 per group); upper scale bar = 2 mm, middle scale bar = 500 µm, lower scale bar = 100 µm. Data were presented as mean ± SEM. Statistical significance was assessed by one-way ANOVA. *p < 0.05, **p < 0.01, ***p < 0.001.

MI is an acute injury of the myocardium; however, myocardial repair after infarction is a chronic process. Therefore, echocardiography was performed on the 28th-day post-MI, which is the healing phase of MI. Regardless of the long-axis mode or short-axis mode, cardiac dysfunction and LV dilatation were significantly ameliorated in the AAV9-Ndufs1 group compared with the AAV9-Ctrl group, as evidenced by an increase in EF and a decrease in LVIDs (Fig. 3a–f). In addition, the biomarkers of HF were significantly enhanced in the hearts of the mice with MI, while Ndufs1 overexpression attenuated the elevation in the mRNA levels of ANP and BNP compared with those of the AAV9-Ctrl-injected MI groups (Supplementary Fig. 4a, b). The compensation of cardiac and pulmonary hypertrophy in response to MI in the Ndufs1-overexpressing mice was attenuated, as demonstrated by the decreases in HW/BW and LW/BW; however, no significant differences in body weights were observed (Supplementary Fig. 4c–e). Compared with the control mice, the cardiac Ndufs1-overexpressing mice displayed a reduced infarct size and myocardial fibrosis, as indicated by Masson trichrome staining (Fig. 3g). Moreover, the above results were confirmed by the attenuated mRNA levels of fibrotic markers such as collagen-1 (Col1a1) and collagen-3 (Col3a1) (Supplementary Fig. 4f, g). These data suggested that cardiac-specific overexpression of Ndufs1 could significantly ameliorate cardiac dysfunction and adverse cardiac remodeling in the healing phase post-MI.

### Upregulation of Ndufs1 expression ameliorates MI/hypoxia-induced mitochondrial morphological and functional aberrations

**Fig. 4:**
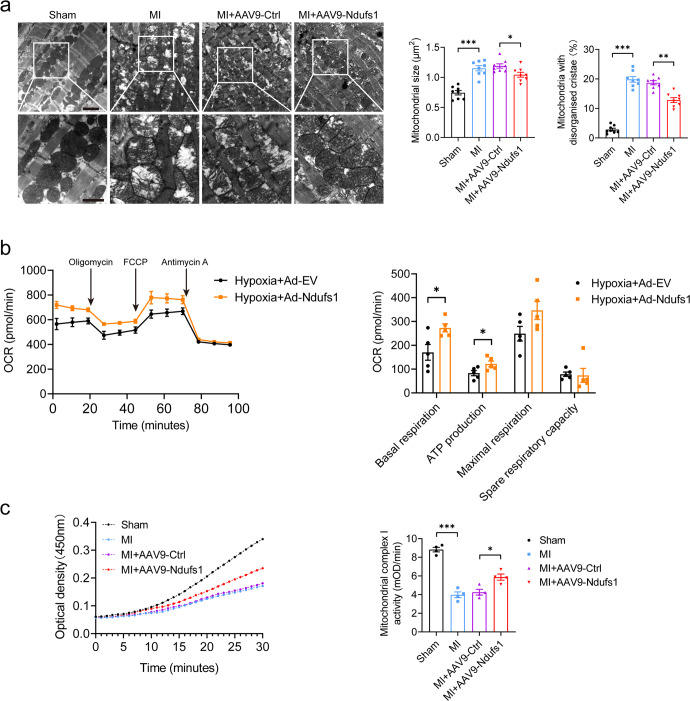
Upregulation of Ndufs1 expression ameliorates MI/hypoxia-induced mitochondrial morphological and functional aberrations. **a** Representative transmission electron microscopy images and quantitative analysis of the mitochondrial size and the proportion of mitochondria with disorganized cristae (*n* = 8 per group); upper scale bar = 2 µm, lower scale bar = 1 µm. **b** Measurements of OCR and quantitative analysis of mitochondrial respiratory function in NRCMs (*n* = 5 per group). **c** Representative absorbance curve with time at OD450 nm and quantitative analysis of mitochondrial complex I activity in NRCMs (*n* = 4 per group). Data were presented as mean ± SEM. Statistical significance was assessed by one-way ANOVA. *p < 0.05, **p < 0.01, ***p < 0.001.

The cardiac contractile function is closely associated with the mitochondrial energy supply to cardiomyocytes. Since Ndufs1 overexpression alleviated MI-induced cardiac dysfunction, we speculated that this effect could be correlated with mitochondrial morphology and functional changes. TEM imaging demonstrated that the hearts of the mice with MI exhibited damaged mitochondria with disorganized cristae compared with the sham controls (Fig. 4a). Additionally, mitochondrial morphology showed distinct heterogeneity in terms of fragmentation and swelling. Interestingly, Ndufs1 overexpression alleviated MI-induced cristae disorder and the appearance of vacuoles. Compared with that of the AAV9-Ctrl group, a decrease in matrix electron density was observed (Fig. 4a). Changes in the morphological structures of organelles are often accompanied by alterations in their functions. Therefore, a mitochondrial respiratory function was examined using a Seahorse extracellular flux analyzer. An adenoviral vector carrying Ndufs1 significantly increased the expression of Ndufs1 in NRCMs (Supplementary Fig. 5a). As expected, compared with cardiomyocytes infected with adenoviral empty vectors (Ad-EV), Ndufs1 overexpression significantly improved mitochondrial respiratory capacity, including basal respiration and ATP production, after hypoxia treatment (Fig. 4b). As the largest among the seven nuclear-encoded core subunits in mitochondrial complex I, Ndufs1 plays a critical role in controlling complex I activity. Indeed, MI-induced deficiency of Ndufs1 was highly associated with a decrease in complex I activity. Compared with AAV9-Ctrl injection, AAV9-Ndufs1 injection significantly improved mitochondrial complex I activity in the hearts of the mice subjected to MI (Fig. 4c). Taken together, our data confirmed that cardiac-specific overexpression of Ndufs1 attenuated MI-induced mitochondrial morphological aberrations and a decrease in complex I activity.

### Cardiac-specific overexpression of Ndufs1 decreases MI-induced oxidative stress and apoptosis

**Fig. 5:**
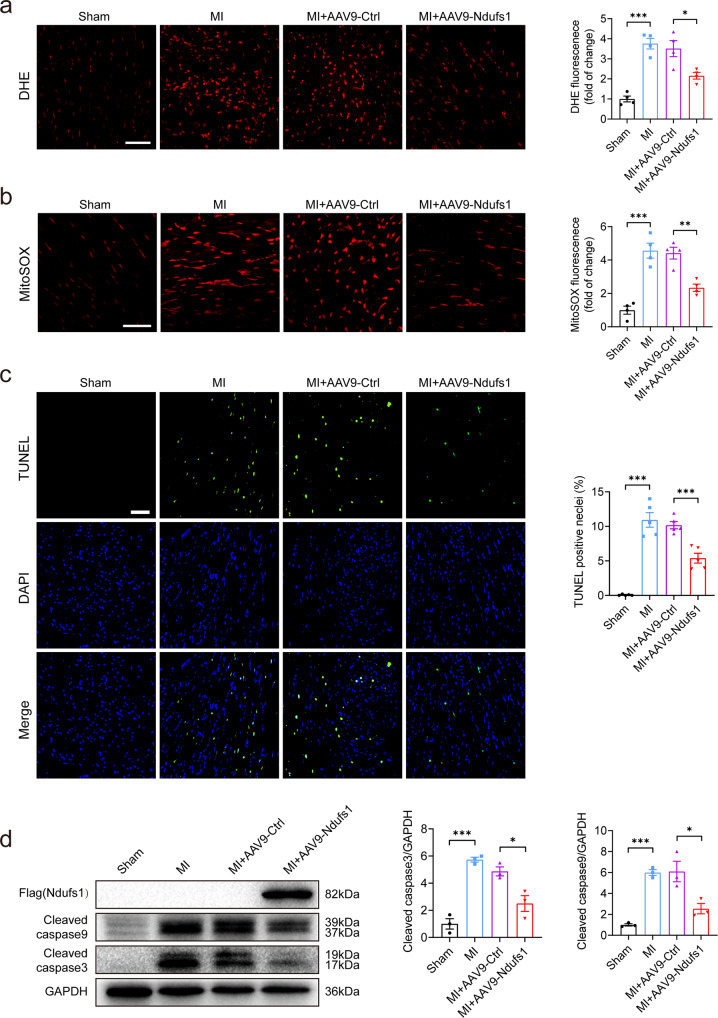
Cardiac-specific overexpression of Ndufs1 decreases MI-induced oxidative stress and apoptosis. **a** Representative confocal microscopy images and quantitative analysis depicting the levels of superoxide anions after DHE staining on the 3rd-day post-MI (*n* = 4 per group); scale bar = 50 µm. **b** Representative confocal microscope images and quantitative analysis depicting the levels of mitochondrial ROS after MitoSOX staining on the 3rd-day post-MI (*n* = 4 per group); scale bar = 50 µm. **c** TUNEL assay by double staining with DAPI (blue) and TUNEL (green) showed apoptotic cells in mouse hearts on the 3rd-day post-MI. Quantification of TUNEL-positive nuclei is shown (*n* = 5 per group); scale bar = 50 µm. **d** Representative blots and quantitative analysis of cleaved caspase 3 and cleaved caspase 9 levels (*n* = 3 in each group). Data were presented as mean ± SEM. Statistical significance was assessed by one-way ANOVA. *p < 0.05, **p < 0.01, ***p < 0.001.

The physiological levels of ROS have an important role in intracellular signaling. However, ROS generation can increase dramatically under some conditions, such as myocardial ischemia, eventually resulting in oxidative stress-induced damage. As anticipated, DHE staining demonstrated an absolute increase in total ROS levels in the hearts of the mice with MI, while the increase was significantly inhibited in the mice overexpressing Ndufs1 (Fig. 5a). Mitochondrial complexes I and III are important sites of ROS production. Compared with the AAV9-Ctrl group, the AAV9-Ndufs1 injection group showed significantly reduced mitochondrial ROS levels, as indicated by MitoSOX staining results (Fig. 5b). Reduced glutathione (GSH) levels are an important marker of intracellular redox status. Oxidation levels were higher in the hearts of the mice with MI, as evidenced by the lower GSH levels, while the high oxidation level was significantly decreased in mouse hearts overexpressing Ndufs1 (Supplementary Fig. 5b). Glutathione peroxidase (GPx) activity also underwent a compensatory increase in the mice with MI, whereas Ndufs1 overexpression decreased enzymatic activity (Supplementary Fig. 5c). Excess ROS levels tend to damage DNA and lead to apoptosis. TUNEL assays revealed that compared with the sham-treated mice, the mice with MI showed apoptosis, which was significantly inhibited upon AAV9-Ndufs1 injection in the rescue experiment (Fig. 5c). Consistently, the amelioration of MI-induced cell apoptosis was further confirmed by a decrease in the levels of cleaved caspase 9 and cleaved caspase 3 (Fig. 5d). In cultured NRCMs, compared with Ad-EV treatment, Ndufs1 overexpression significantly alleviated hypoxia-induced cell apoptosis (Supplementary Fig. 5d). The phenotypic analysis provided in vivo and in vitro evidence for Ndufs1-mediated attenuation of MI/hypoxia-induced mitochondrial ROS production and subsequent cell apoptosis.

### Sp1 binds to the Ndufs1 promoter region and positively regulates its transcription

**Fig. 6:**
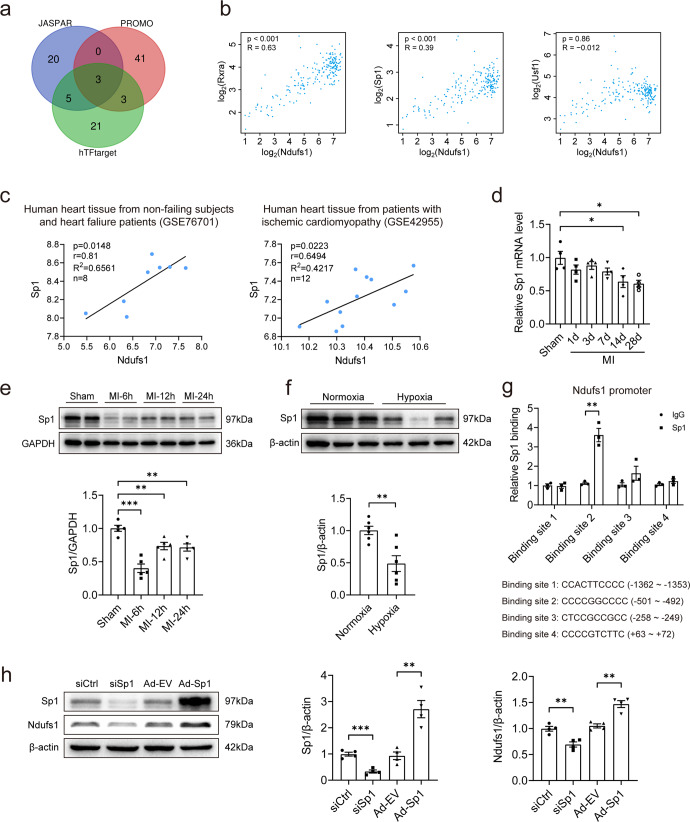
Sp1 binds to the Ndufs1 promoter region and positively regulates its transcription. **a** Venn diagram showing the common transcription factors identified in the three databases. **b** Scatter plots showing the correlation of mRNA levels between Nudfs1 and candidate transcription factors in human left ventricular samples through the GEPIA website with data from the GTEx database. **c** Scatter plots showing the correlation of mRNA levels of Nudfs1 and Sp1 in human heart tissue based on two datasets from the GEO database. **d** qRT-PCR analysis of Sp1 levels in mouse heart tissues at the indicated time points post-MI (*n* = 4 per group). **e** Representative blots and quantitative analysis of Sp1 expression in mouse heart tissues at the indicated time points post-MI (*n* = 5 per group). **f** Representative blots and quantitative analysis of Sp1 expression in NRCMs with or without hypoxia (*n* = 6 per group). **g** ChIP assay and qRT-PCR analysis showing Sp1 binding to the Ndufs1 promoter in HL-1 cells (*n* = 3 per group). **h** Representative blots and quantitative analysis of Sp1 and Ndufs1 expression in HL-1 cells (*n* = 4 per group). Data were presented as mean ± SEM. Statistical significance was assessed by one-way ANOVA. *p < 0.05, **p < 0.01, ***p < 0.001.

Given that Ndufs1 has profound effects on mitochondrial function and cell survival in MI, we examined the upstream regulator of Ndufs1. By querying three databases, we identified three potential transcription factors for human Ndufs1 (Fig. 6a). Correlations of the above three potential transcription factors with Ndufs1 were analyzed separately in human LV samples from the GEPIA (gene expression profiling interactive analysis) database. This analysis suggested that Rxra and Sp1 were the most promising transcription factor candidates (Fig. 6b). Upon further analysis of the potential binding sites of Rxra and Sp1 in the Ndufs1 promoter region using the JASPAR database, Sp1 was identified as a putative transcription factor of Ndufs1 (Supplementary Fig. 6a, b). Next, we evaluated the mRNA level of Sp1 using a public microarray dataset from the GEO database. Scatter plot analysis showed a significant positive correlation between Ndufs1 and Sp1 mRNA levels in NF subjects and HF patients; similar results were obtained in patients with ischemic cardiomyopathy (Fig. 6c). Furthermore, Sp1 mRNA levels were significantly decreased in the mouse hearts on the 14th-day and 28th-day post-MI compared with those of the sham group (Fig. 6d). Notably, in the initial 6 h post-MI, the protein level of myocardial Sp1 was reduced significantly (Fig. 6e). The above results were also observed in hypoxia-treated NRCMs (Fig. 6f). In addition, the ChIP assay demonstrated the direct binding of Sp1 to the putative site in the Ndufs1 promoter region in HL-1 cells (Fig. 6g). Sp1 knockdown significantly decreased the expression of Ndufs1 protein, while Sp1 overexpression led to a significant upregulation of Ndufs1 expression (Fig. 6h). Taken together, our data suggested that decreased Ndufs1 in the heart tissue of HF patients and mice post-MI was at least in part due to the decrease in the expression of Sp1.

### Sp1 overexpression alleviates hypoxia-induced mitochondrial dysfunction, fibrosis, and apoptosis by upregulating Ndufs1 expression

**Fig. 7:**
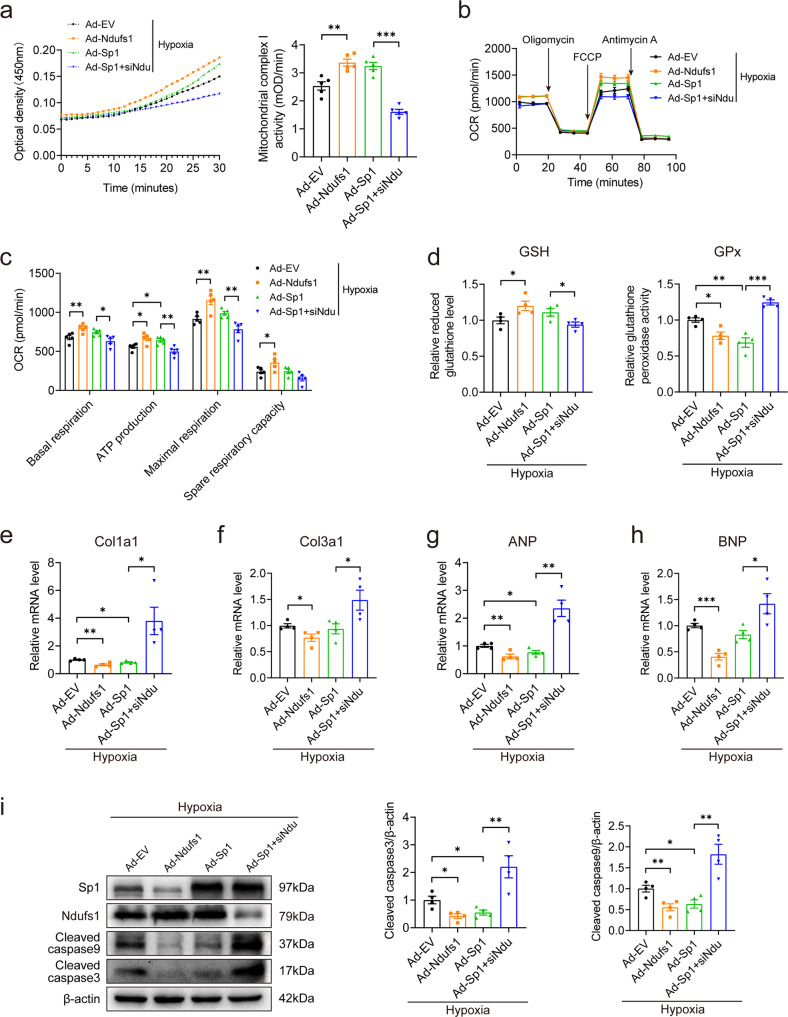
Sp1 overexpression alleviates hypoxia-induced mitochondrial dysfunction, fibrosis, and apoptosis by upregulating Ndufs1 expression. **a** Representative absorbance curve with time at OD450 nm and quantitative analysis of mitochondrial complex I activity in HL-1 cells (*n* = 5 per group). **b**, **c** Measurements of OCR and quantitative analysis of mitochondrial respiratory function in HL-1 cells (*n* = 5 per group). **d** GSH levels and GPx activity in NRCMs (*n* = 4 per group). **e**–**h** qRT-PCR analysis of Col1a1, Col3a1, ANP, and BNP levels in NRCMs (*n* = 4 per group). **i** Representative blots and quantitative analysis of cleaved caspase 3 and cleaved caspase 9 expression in NRCMs (*n* = 4 per group). Data were presented as mean ± SEM. Statistical significance was assessed by one-way ANOVA. *p < 0.05, **p < 0.01, ***p < 0.001.

An adenovirus carrying the Sp1 construct was used, and the effects of overexpression were demonstrated in NRCMs (Supplementary Fig. 6c). Upregulation of Sp1 expression significantly elevated the activity of mitochondrial complex I in HL-1 cells subjected to hypoxia, while this effect was inhibited by Ndufs1 knockdown (Fig. 7a). Upregulation of Sp1 expression or upregulation of Ndufs1 expression alleviated hypoxia-induced mitochondrial respiratory dysfunction in HL-1 cells, while the protective effect of upregulated Sp1 expression was lost upon Ndufs1 knockdown (Fig. 7b, c). Similarly, compared with Ad-EV treatment, Sp1 overexpression or Ndufs1 overexpression attenuated hypoxia-induced elevation in intracellular oxidation levels, the level of fibrotic markers, and the level of HF markers in NRCMs, whereas the protective effect of Sp1 was inhibited by Ndufs1 knockdown, as evidenced by GSH levels, GPx activity, and the mRNA levels of Col1a1, Col3a1, ANP, and BNP (Fig. 7d–h). Furthermore, Western blotting showed that Ndufs1 knockdown suppressed the beneficial effect of Sp1 overexpression in reducing hypoxia-induced apoptosis in NRCMs (Fig. 7i). Thus, these results revealed that the protective effects of upregulated Sp1 expression on hypoxia-induced NRCMs were mediated, at least in part, through Ndufs1.

## Discussion

**Fig. 8:**
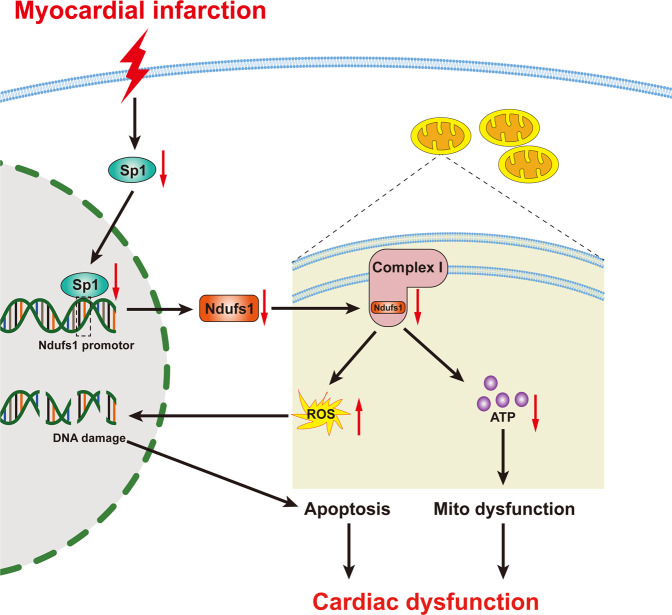
Schematic diagram depicting the mechanisms by which MI-induced reduction of Ndufs1 leads to cardiac dysfunction by exacerbating mitochondrial dysfunction and apoptosis. MI-induced Sp1 reduction decreases Ndufs1 transcription in the nucleus. Ndufs1 reduction in mitochondrial complex I increase mitochondrial ROS generation and decreases ATP production, which causes cell apoptosis and mitochondrial dysfunction, respectively, leading to cardiac dysfunction.

In the present study, we found that Ndufs1 expression was significantly decreased in the hearts of heart failure patients and mice after MI. Ndufs1 overexpression mediated by AAV9 attenuated cardiac dysfunction and myocardial fibrosis during the healing phase of MI. More specifically, upregulation of Ndufs1 expression reduced MI-induced aberrations in the mitochondrial cristae and improved mitochondrial respiratory function by enhancing mitochondrial complex I activity. The restoration of Ndufs1 expression also decreased MI-induced ROS generation and ROS-related apoptosis. Furthermore, decreased Ndufs1 in the hearts of the mice with MI was caused by the reduction in Sp1 expression. Upregulated Sp1 expression partially alleviated hypoxia-induced oxidative stress and mitochondrial dysfunction. Therefore, Ndufs1 may be a prospective therapeutic target for treating cardiac dysfunction caused by MI (Fig. 8).

Heart failure after MI is characterized by hypoxia-induced cardiomyocyte death and dysfunction of the surviving cardiomyocytes^5,28^. Therefore, effective therapeutic measures are needed to reduce cell death caused by blood flow blockage and improve the contractile function of the surviving cardiomyocytes. Based on the characteristics of rhythmic systole-diastole of cardiomyocytes and the properties of intracellular redox state changes during MI, mitochondrial energy supply and ROS generation aroused our interest. ATP production by oxidative phosphorylation in mitochondrial ETCs is accompanied by ROS generation in cardiomyocytes. Complex I is the initiating complex enzyme and the main site of ROS generation in ETCs^17,18^. Complex I deficiency or decreased activity directly correlates with reduced mitochondrial respiratory function^20,29^. Most studies on complex I activity focus on the core subunits of complex I^30–32^. Ndufs2 (NADH:ubiquinone oxidoreductase core subunit S2) is a core subunit in complex I encoded by nuclear genes. Disruption of Ndufs2 significantly decreased cell growth in vitro, complex I-specific respiration, and the ATP pool and increased ROS generation and apoptosis^29,33,34^. In addition, in primary skin fibroblasts obtained from Leigh syndrome patients with isolated complex I deficiency, abnormalities in mitochondrial membrane potential, ROS levels, and intracellular ATP content were closely associated with mutations in the nuclear-encoded complex I genes, including Ndufs7, Ndufs8, and Ndufv1^30^. A recent study found that Ndufs1 expression was also decreased in a mouse model of pressure overload-induced myocardial hypertrophy^35^. However, little is known about the role of Ndufs1 and its functional mechanisms in cardiac ischemic injury.

First, we started from the available public database and found that the expression of Ndufs1 was decreased in the left heart of patients with heart failure. Second, we found that Ndufs1 expression was decreased in patients with heart failure, in mice with myocardial infarction, and in hypoxia-treated NRCMs. Third, to achieve prolonged cardiac-specific high expression of Ndufs1, we used adeno-associated virus serotype 9 with a cardiac-specific promoter, cTnT, as the vector in rescue experiments. The MI procedure was performed only after it became clear that Ndufs1 was efficiently expressed in the mouse heart. Interestingly, Ndufs1 overexpression effectively alleviated cardiac dysfunction in the healing phase of MI and reduced infarct size and myocardial fibrosis but had no significant beneficial effects on cardiac function and structure in the acute phase of MI. At the organelle level, Ndufs1 overexpression, to some extent, rescued the morphological abnormalities in mitochondrial cristae and mitochondrial respiratory dysfunction. One possible reason for this discrepancy between the acute and healing phases might be that Ndufs1 overexpression positively affected cellular substructures such as mitochondria in the early stages of MI. However, this effect was not strong enough to be reflected in phenotypes such as cardiac function in time. As LV remodeling progresses after MI, this beneficial effect on mitochondria persists and is reflected in protecting cardiac function and structure. Furthermore, hypoxia impaired the normal oxygen consumption of cardiomyocytes. Upregulation of Ndufs1 expression alleviated this damage, which could be explained by improved complex I activity. Increased ROS generation can promote apoptosis through multiple pathways^36,37^. This study focused on direct damage to DNA in the nucleus due to elevated ROS levels and the activation of caspases, apoptosis-related proteins in the mitochondrial pathway. Zou et al. also showed that ROS generation was significantly elevated after Ndusf1 silencing, and Ndufs1 overexpression attenuated Ang II-induced ROS generation and mitochondrial dysfunction in cardiomyocytes^35^. This evidence suggests that Ndufs1 may have similar mechanisms of action in various cardiovascular diseases and is a valuable therapeutic target.

What causes the decreased expression of Ndufs1 in the myocardium after MI remains unknown. Given that the interaction between promoters and transcription factors is one of the most common methods to regulate gene expression in nature, a ChIP experiment was performed to explore the reason for the reduced expression of Ndufs1 in MI. Sp1, a broad-spectrum transcription factor, regulates the transcription of multiple genes in several disease models^38–40^. A previous study demonstrated that Sp1 expression was downregulated in the mouse heart subjected to ischemia/reperfusion (I/R) injury^41^. Sp1 overexpression reduced infarct size and exerted an antiapoptotic effect in I/R models, which is similar to our results^41^. Our results confirmed that Sp1 was the upstream transcription factor of Ndufs1. Interestingly, in the MI mouse model, Ndufs1 protein levels gradually decreased from day one post-MI, while the protein expression of Sp1 significantly decreased to the lowest level at 6 h post-MI. There were several possible reasons for the incomplete concordance between the expression of the two proteins. Most likely, the MI-induced decrease in Sp1 expression led to a decrease in Ndufs1 mRNA, which took a certain time for translation into protein in vivo. In addition, the MI-induced reduction of Ndufs1 at the transcriptional level lagged at the translational level, which could be explained by the presence of posttranslational modifications in Ndufs1, leading to its protein degradation. A previous study reported increased levels of Ndufs1 acetylation in the post-MI mouse gastrocnemius, which may be related to Ndufs1 degradation and its reduced function^42^. Additionally, we found that overexpression of Ndufs1 in cardiomyocytes, in turn, inhibited Sp1 expression, as shown in Fig. 7i. Given that Sp1 acts as a transcription factor to activate the expression of Ndufs1, after the overexpression of Ndufs1, there may be a feedback loop to inhibit the expression of Sp1, which normalizes the intracellular Ndufs1, thereby maintaining the normal physiological activity of cardiomyocytes.

Previous studies on Ndufs1 were mainly performed using primary cells or in cell lines^20,43^. Our study provides further validation based on in vivo experiments with animal models. However, there are several limitations to the present study. First, due to ethical requirements, we were unable to verify the functional role of Ndufs1 in humans. All in vivo experimental results were validated in the mouse model. An advantage is that the results may be more reproducible because individual differences are smaller in mice than in humans. Second, although Ndufs1 is critical in MI, its change in expression does not fully explain the alterations in cardiac function. Other regulatory modalities may influence its function, which needs to be explored by further studies. Despite these limitations, our study offers a new perspective on the role of Ndufs1 in HF after MI.

Given the excellent properties of adeno-associated viruses, including prolonged-expression and low immunogenicity, AAV-mediated gene therapy has been widely used in preclinical and clinical studies^44–46^. To date, over 140 interventional clinical trials involving AAV have been registered, and at least two vectorized AAV serotypes have received regulatory approval for commercial use in patients^47^. Since 2012, three AAV-based gene therapeutic drugs have received approval from the European Medicines Agency and the United States Food and Drug Administration for the treatment of related diseases^48^. We believe this AAV9-mediated Ndufs1 gene therapy approach can be applied for patients with post-MI heart failure and other cardiovascular diseases. Clinically, AAV administration could be used to alleviate cardiac dysfunction or delay its progression to heart failure, which would be extremely beneficial for patients with MI. This treatment may prevent patients from being hospitalized and substantially improve their quality of life.

In summary, cardiac-specific Ndufs1 overexpression could effectively alleviate cardiac dysfunction in the healing phase of MI, which may be attributed to improved mitochondrial function and reduced ROS generation. Our study suggests that Ndufs1 overexpression through the AAV9 method may be a promising therapeutic strategy for heart failure after myocardial infarction.

## Supplementary information

Supplemental Information
