# Supplementary material for: Cardiac-specific overexpression of Ndufs1 ameliorates cardiac dysfunction after myocardial infarction by alleviating mitochondrial dysfunction and apoptosis
Source: Exp Mol Med. 2022 Jul 11;54(7):946–60. doi: 10.1038/s12276-022-00800-5 (PMC9355970; doi:10.1038/s12276-022-00800-5)
Supplement: Supplementary file 1 — Supplemental Information [file 12276_2022_800_MOESM1_ESM.pdf]

Supplemental Information

Cardiac-specific overexpression of Ndufs1 ameliorates cardiac dysfunction after myocardial infarction by alleviating mitochondrial dysfunction and apoptosis

Bingchao Qi,[1,3] Liqiang Song[2,3], Lang Hu,[1,3] Dong Guo,[1] Gaotong Ren,[1] Tingwei Peng,[1] Mingchuan Liu,[1] Yexian Fang,[1] Chunyu Li,[1] Mingming Zhang,[1] and Yan Li.[1]

[1]Department of Cardiology, Tangdu Hospital, Fourth Military Medical University, Xi'an, 710038, People's Republic of China.

[2]Department of Pulmonary and Critical Care Medicine, Xijing Hospital, Fourth Military Medical University, Xi'an, 710032, People's Republic of China.

[3]These authors contributed equally.

Address correspondence to:

Prof. Yan Li, profleeyan@163.com.

Prof. Mingming Zhang, winterzhang3@163.com.

Supplementary Figures

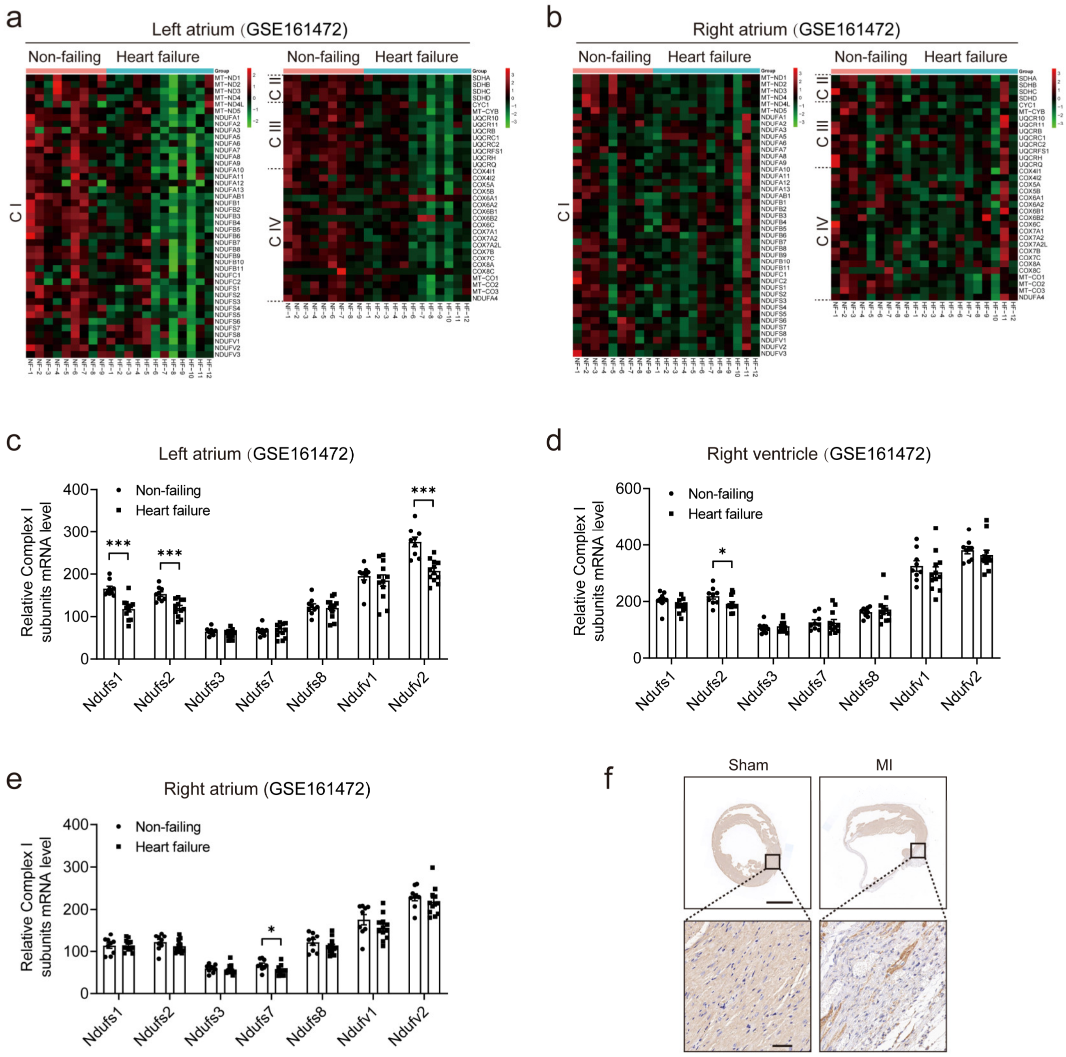

Supplementary Fig. 1 Ndufs1 expression is decreased in the myocardium of heart failure patients and post-MI mice

a, **b** Heat map of RNA-seq analysis for mitochondrial genes belonging to complex I (C I), complex II (C II), complex III (C III), and complex IV (C IV) in the left atrium samples and right atrium samples of non-failing subjects (n = 9) and heart failure patients (n = 12). **c-e** Mitochondrial Complex I subunits mRNA levels in three heart chambers of non-failing subjects (n = 9) and heart failure patients (n = 12) based on the RNA-seq data. **f** Representative immunohistochemical staining images of Ndufs1 in the

hearts of sham mice and mice on the 28th-day post-MI; upper scale bar = 2 mm, lower scale bar = 50 μm. Data were presented as means ± SEM. Statistical significance was assessed by one-way ANOVA. *$p < 0.05$, **$p < 0.01$, ***$p < 0.001$.

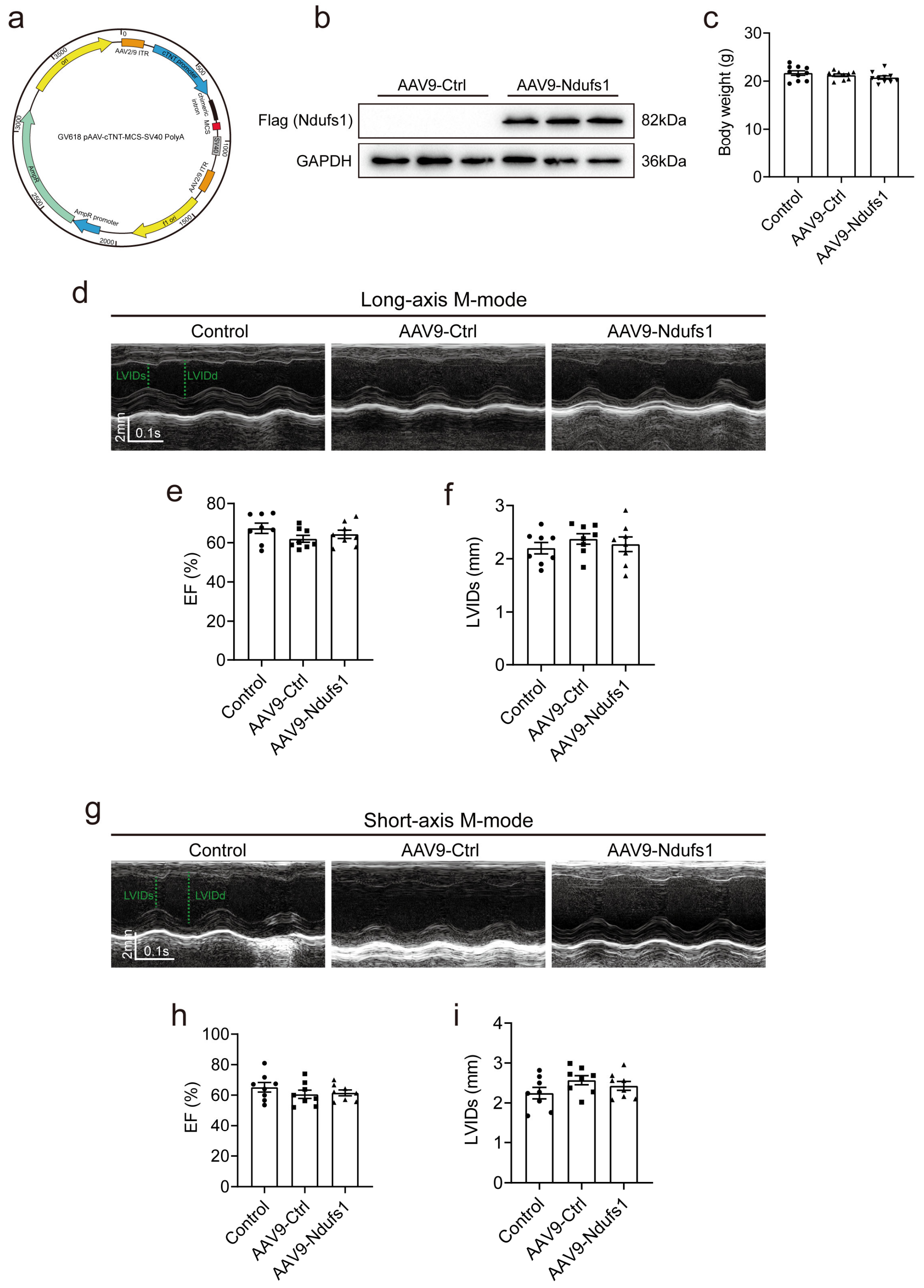

Supplementary Fig. 2 Cardiac-specific overexpression of Ndufs1 has no effect on cardiac function in C57BL/6J mice

a Schematic diagram of the adeno-associated virus serotype 9 (AAV9) vector under the control of a cardiac troponin T promoter (cTnT) used in this study. **b** Representative blots showing overexpression effect by AAV9-Ndufs1 infection in hearts of C57BL/6 J mice. **c** Body weight of mice at 4 weeks after AAV injection (n = 10 per group). **d** Representative long-axis M-mode echocardiograph images at 4 weeks after AAV injection. LVIDs, left ventricular systolic internal dimension; LVIDd, left ventricular diastolic internal dimension. **e**, **f** Echocardiograph analysis in the long-axis M-mode (n = 8 per group). EF, ejection fraction. **g** Representative short-axis M-mode echocardiograph images at 4 weeks after AAV injection. **h**, **i** Echocardiograph analysis in the short-axis M-mode (n = 8 per group). Data were presented as means ± SEM. Statistical significance was assessed by one-way ANOVA.

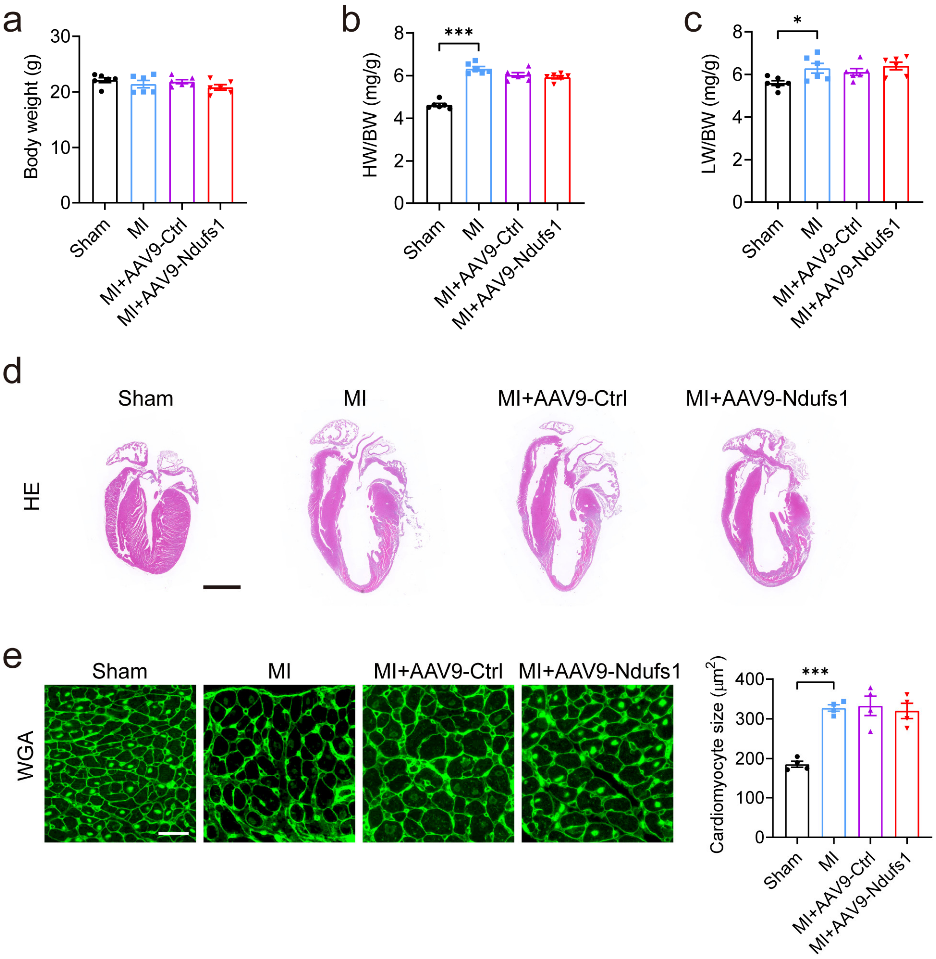

Supplementary Fig. 3 Cardiac-specific overexpression of Ndufs1 has no effect on compensatory myocardial hypertrophy in the acute phase of MI

a-c Body weight, heart weight/body weight (HW/BW), and lung weight/body weight (LW/BW) on the 3rd-day post-MI (*n* = 6 per group). **d** Hematoxylin and eosin (HE) staining showed the gross morphology of hearts on the 3rd-day post-MI; scale bar = 2 mm. **e** Representative images of wheat germ agglutinin (WGA) staining and quantitative analysis of the cross-sectional area of cardiomyocytes on the 3rd day post-MI (*n* = 4 per group); scale bar = 30 μm. Data were presented as means ± SEM. Statistical significance was assessed by one-way ANOVA. *$p < 0.05$, ***$p < 0.001$.

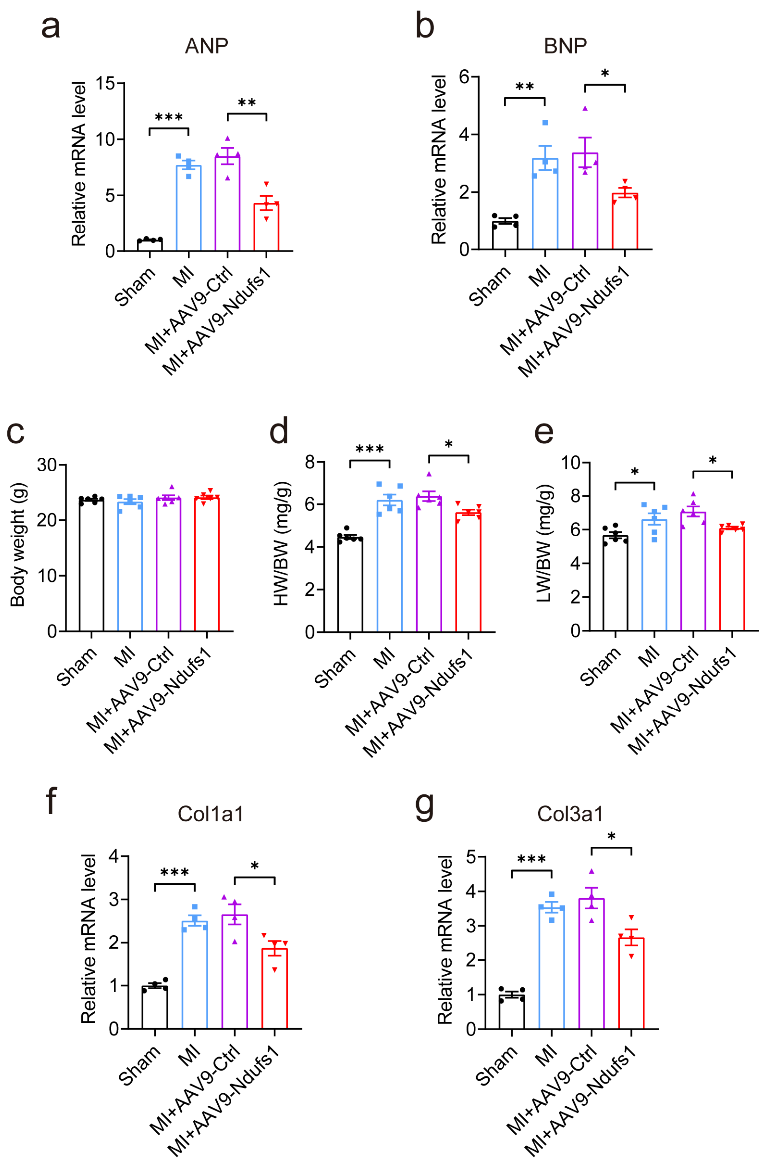

Supplementary Fig. 4 Cardiac-specific overexpression of Ndufs1 significantly decreases the makers of heart failure and myocardial fibrosis in the healing phase of MI

a, **b** qRT-PCR analysis of the makers of heart failure, including ANP and BNP, at 4 weeks after MI (*n* = 4 per group). **c-e** Body weight, HW/BW, and LW/BW at 4 weeks after MI (*n* = 6 per group). **f**, **g** qRT-PCR analysis of the makers of myocardial fibrosis, including Col1a1 and Col3a1, at 4 weeks after MI (*n* = 4 per group). Data were presented as means ± SEM. Statistical significance was assessed by one-way ANOVA.

*$p < 0.05$, **$p < 0.01$, ***$p < 0.001$.

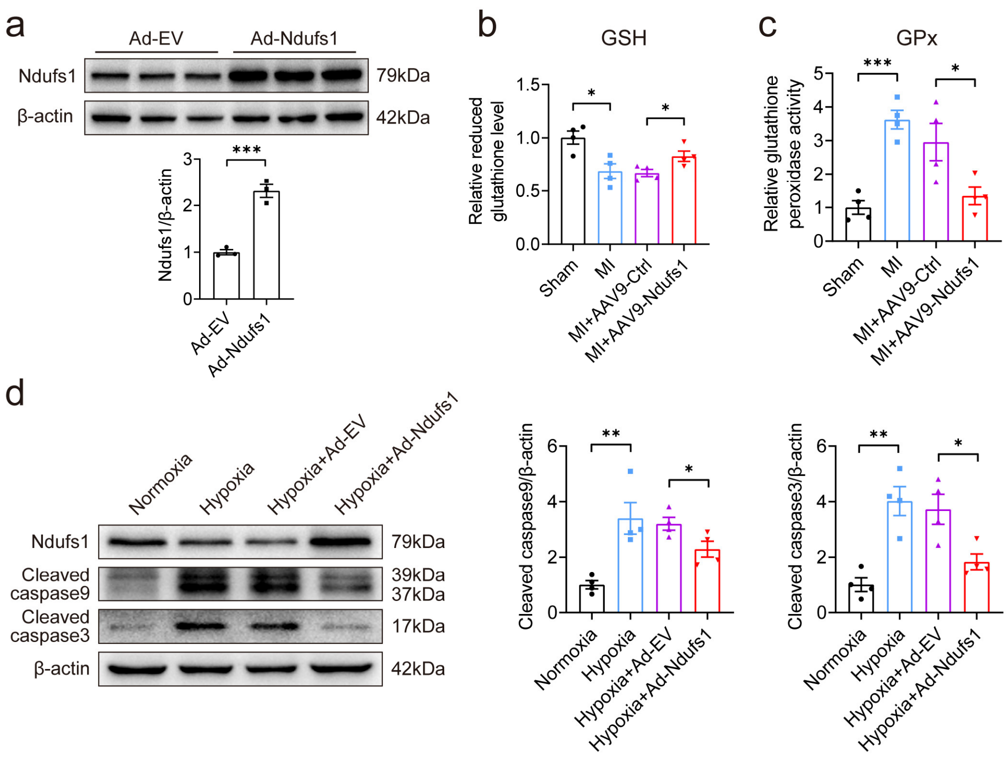

Supplementary Fig. 5 Upregulation of Ndufs1 decreases MI-induced oxidative stress and hypoxia-induced apoptosis

a Representative blots and quantitative analysis of overexpression effect by Ad-Ndufs1 infection in NRCMs ($n = 3$ per group). **b**, **c** GSH level and GPx activity in mouse heart tissues ($n = 4$ per group). **d** Representative blots and quantitative analysis of cleaved caspase 9 and cleaved caspase 3 levels in NRCMs ($n = 4$ per group). Data were presented as means ± SEM. Statistical significance was assessed by one-way ANOVA. *$p < 0.05$, **$p < 0.01$, ***$p < 0.001$.

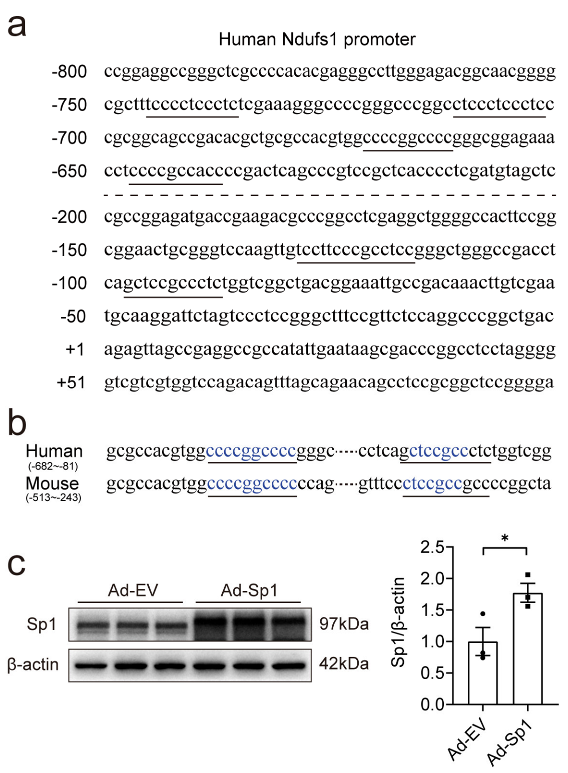

Supplementary Fig. 6 Potential binding sites for the transcription factor Sp1

a Potential binding sites for Sp1 in the human Ndufs1 promoter region. **b** Conserved putative Sp1 binding sites of Ndufs1 promoter region in human and mouse. **c** Representative blots and quantitative analysis of overexpression effect by Ad-Sp1 infection in NRCMs (n = 3 per group). Statistical significance was assessed by Student's t-test. *$p < 0.05$.

Supplementary Tables

Supplementary Table 1

The primer sequences used for quantitative PCR were as follows:

Species	Gene	Forward primer (5'-3')	Reverse primer (5'-3')
Human	*Ndufs1*	TTAGCAAATCACCCATTGGACTG	CCCCTCTAAAAATCGGCTCCTA
Human	*β-actin*	CCTGGCACCCAGCACAAT	GGGCCGGACTCGTCATAC
Mouse	*Ndufs1*	AGGATATGTTCGCACAACTGG	TCATGGTAACAGAATCGAGGGA
Mouse	*Ndufs2*	CAGCCAGATATTGAATGGGCA	TGTTGGTCACCGCTTTTTCCT
Mouse	*Ndufs3*	TGGCAGCACGTAAGAAGGG	CTTGGGTAAGATTTCAGCCACAT
Mouse	*Ndufs7*	GTTCATCAGAGTGTAGCCACTG	CAGGCCGAAGGTCATAGGC
Mouse	*Ndufs8*	AGTGGCGGCAACGTACAAG	TCGAAAGAGGTAACTTAGGGTCA
Mouse	*Ndufv1*	TTTCTCGGCGGGTTGGTTC	GGTTGGTAAAGATCCGGTCTTC
Mouse	*Ndufv2*	GCAAGGAATTTGCATAAGACAGC	TAGCCATCCATTCTGCCTTTG
Mouse	*Col1a1*	GGAGACAGGTCAGACCTGTGTG	CAGCTGGATAGCGACATCGGC
Mouse	*Col3a1*	CTGTAACATGGAAACTGGGGAAA	CTGTAACATGGAAACTGGGGAAA
Mouse	*ANP*	GCTTCCAGGCCATATTGGAGCA	TCTCTCAGAGGTGGGTTGACCT
Mouse	*BNP*	ATGGATCTCCTGAAGGTGCTGT	GCAGCTTGAGATATGTGTCACC
Mouse	*Sp1*	GCCGCCTTTTCTCAGACTC	TTGGGTGACTCAATTCTGCTG
Mouse	*GAPDH*	GGCTGCCCAGAACATCAT	CGGACACATTGGGGGTAG
Rat	*Ndufs1*	CCAAAGTAGCAGTGACACCTCC	CAACATCATCATATCGAACCAG
Rat	*Col1a1*	ATCAGCCCAAACCCCAAGGAGA	CGCAGGAAGGTCAGCTGGATAG
Rat	*Col3a1*	TGCCATTGCTGGAGTTGGA	GAAGACATGATCTCCTCAGTGTTGA
Rat	*ANP*	GGAGCCTGCGAAGGTCAA	TATCTTCGGTACCGGAAGCTGT
Rat	*BNP*	CAGTCAGTCGCTTGGGCTGT	GCAGAGTCAGAAGCCGGAGT
Rat	*β-actin*	TGTCACCAACTGGGACGATA	GGGGTGTTGAAGGTCTCAAA

Supplementary Table 2

Primary antibodies used for western blotting and immunohistochemistry were as follows:

Antibody	Source	Company	Catalogue Number
Ndufs1 (WB, IHC)	Rabbit	Abcam	ab169540
GAPDH (WB)	Rabbit	Proteintech	10494-1-AP
β-actin (WB)	Rabbit	Proteintech	20536-1-AP
Hif1α(WB)	Rabbit	Proteintech	20960-1-AP
DDDDK tag (WB)	Rabbit	Abcam	ab205606
Cleaved caspase-3 (WB)	Rabbit	CST	9664
Cleaved caspase-9 (WB)	Rabbit	CST	20750
Sp1 (ChIP)	Rabbit	Active Motif	39058
Sp1 (WB)	Rabbit	Proteintech	21962-1-AP

Supplementary Table 3

siRNA sequences of primers were as follows:

Target	siRNA sense sequence (5'-3')	siRNA sense sequence (5'-3')
Ndufs1	GCAUGCAAAUCCCUCGAUUTT	AAUCGAGGGAUUUGCAUGCTT
Sp1	UGAGAACAGCAACUCCTT	GGAGUUGUUGCUGUUCUCAUU
Control	UUCUCCGAACGUGUCACGUTT	ACGUGACACGUUCGGAGAATT
